# Risk factors of post-COVID-19 symptoms, a cross-sectional study in patients with asthma and chronic obstructive lung disease

**DOI:** 10.1038/s41533-026-00570-x

**Published:** 2026-09-29

**Authors:** Agnes Andreason, Björn Ställberg, Karin Lisspers, Anna Nager, Hanna Sandelowsky, Josefin Sundh, Gabriella Eliason, Scott Montgomery, Christer Janson, Marta A. Kisiel

**Affiliations:** 1 Department of Medical Sciences, Occupational and Environmental Medicine, Uppsala University, Uppsala, Sweden; 2 Department of Public Health and Caring Sciences, General Practice, Uppsala University, Uppsala, Sweden; 3 Department of Neurobiology, Care Sciences and Society, Division of Family Medicine and Primary Care, Karolinska Institutet, Stockholm, Sweden; 4 Department of Respiratory Medicine, Faculty of Medicine and Health, Örebro University, Örebro, Sweden; 5 Clinical Epidemiology and Biostatistics, School of Medical Sciences, Faculty of Medicine and Health, Örebro University, Örebro, Sweden; Division of Clinical Epidemiology, Department of Medicine, Karolinska Institutet, Stockholm, Sweden; Department of Epidemiology and Public Health, University College London, London, UK; 6 Department of Medical Sciences, Respiratory, Allergy and Sleep Research, Uppsala University, Uppsala, Sweden

**Keywords:** Diseases, Health care, Medical research, Risk factors

## Abstract

Post-COVID-19 symptoms are common following COVID-19, but risk factors of persistent symptoms in patients with asthma and chronic obstructive lung disease (COPD) remain unclear. We aimed to assess the frequency and risk factors of post-COVID-19 symptoms in patients with asthma and COPD. In this cross-sectional study, we used data from the Swedish PRAXIS study collected in 2022. The cohort included randomly selected adult patients with a diagnosis of asthma or COPD in medical records from central Sweden’s primary and secondary care. Participants completed a comprehensive questionnaire, and their responses were analysed. We used logistic regression and adjusted for age and lung disease diagnoses. Among 494 patients with confirmed COVID-19, 59% (289/494) reported post-COVID-19 symptoms. Risk factors for post-COVID-19 symptoms included severe acute infection (adjusted odds ratio aOR: 8.91, 95% confidence interval [CI]: 3.49–22.8), higher age (aOR:1.25, 95% CI: 1.09–1.45), female sex (aOR: 1.52, 95% CI: 1.04–2.21), and obesity [BMI ≥ 30] (aOR: 2.24, 95% CI: 1.44–3.47)). Anxiety/depression (aOR:1.79, 95% CI: 1.13–2.80) and chronic pain (aOR: 3.61, 95% CI: 1.95–6.69) were also significant risk factors. Patients with asthma or COPD had a high prevalence of post-COVID-19 symptoms compared to other studies. Several demographic and clinical factors were associated with post-COVID-19 symptoms, which could help identify patients at increased risk.

## Introduction

Understanding the long-term consequences of COVID-19 in patients with asthma and chronic obstructive lung disease (COPD) remains challenging. Few studies have focused on persistent symptoms, post-COVID-19 condition, in patients with asthma or COPD.

Asthma and COPD are common obstructive lung diseases and affect 8–10% of the population globally. Exacerbations of these conditions are frequently triggered by infections. Both COPD and asthma may increase susceptibility to respiratory infections, including COVID-19, due to their shared pathophysiological traits^1,2^.

Post-COVID-19 condition is defined by the National Institute for Health and Care Excellence (NICE) as “Signs and symptoms that develop during or after an infection consistent with COVID‑19, continue for more than 12 weeks and are not explained by an alternative diagnosis. It usually presents with clusters of symptoms, often overlapping, which can fluctuate and change over time and can affect any system in the body. Post‑COVID‑19 syndrome may be considered before 12 weeks while the possibility of an alternative underlying disease is also being assessed”^3^.

Post-COVID-19 condition is heterogeneous and may include respiratory, neurological, and systemic symptoms, with fatigue, dyspnea, cognitive difficulties, and musculoskeletal symptoms^4,5^. Factors associated with an increased risk of developing post-COVID-19 condition are female sex, older age, higher body mass index, smoking, pre-existing comorbidities, and greater severity of the acute COVID-19 infection^6,7^.

The pathophysiology of post-COVID-19 condition is likely to be multifactorial and remains incompletely understood. Several mechanisms have been proposed, including viral persistence, chronic inflammation, immune dysregulation, endothelial dysfunction, and metabolic or mitochondrial abnormalities^8^. Mechanisms particularly relevant to persistent respiratory symptoms include ongoing inflammation, endothelial and microvascular injury, and impaired metabolic and mitochondrial function. These processes may contribute to prolonged dyspnoea, exercise intolerance, and fatigue^9,10^.

The prevalence of post-COVID-19 condition has been estimated to be as high as 42%^11^. While the exact prevalence of post-COVID-19 condition in Sweden remains unclear, a registry-based study found that 2% of COVID-19 cases were affected^12^. Recent evidence suggests that asthma and COPD are associated with an increased risk of developing post-COVID-19. A systematic review and meta-analysis reported 41% higher odds of post-COVID-19 in individuals with pre-existing asthma and 32% higher odds in those with COPD^13^. Knowledge on characteristics and risk factors is needed to target preventive and management strategies for post-COVID-19. We aim to evaluate the frequency and risk factors of post-COVID-19 symptoms in a Swedish cohort of asthma and COPD patients.

## Materials and methods

### Study design

This cross-sectional study used questionnaire data from the Swedish PRAXIS study, collected in 2022. The cohort consisted of randomly selected patients aged 18–75 years, when recruited, from primary and secondary care settings, with physician-diagnosed asthma or COPD (ICD-10 codes J44 and J45) in their medical records. Diagnoses were established in routine clinical practice according to applicable Swedish clinical guidelines, which are aligned with recommendations from the Global Initiative for Asthma (GINA) and the Global Initiative for Chronic Obstructive Lung Disease (GOLD)^1,2^.

The initial cohorts, recruited in 2005, 2014, and 2015, included patients from primary healthcare centres and hospitals across eight regions in central Sweden^14,15^.

In the spring of 2022, a follow-up questionnaire (see Supplementary Table 1), study information, and a consent form, which formed the basis for this study, were sent by post to all patients who initially participated and were still alive and had a known address (Supplementary Fig. 1). In total, 1513 patients with COPD and 1787 patients with asthma answered the questionnaire.

### COVID-19

**Fig. 1:**
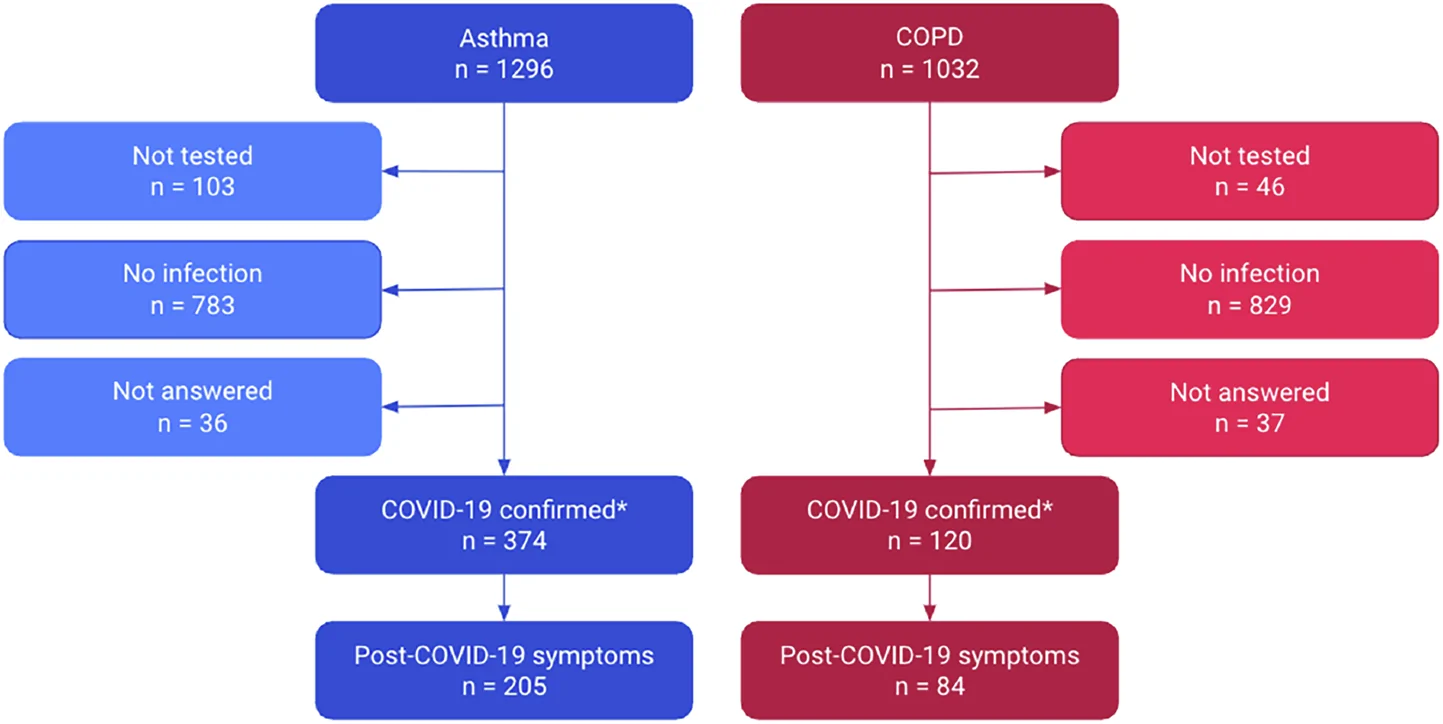
Flow chart of the study population. Notes: *Patients reported confirmed with polymerase chain reaction, antigen test, antibody test, doctor /nurse diagnosis.

Patients with asthma or COPD were considered to have a confirmed COVID-19 infection if they reported any of the following: a positive polymerase chain reaction (PCR) test, a positive antigen test, a positive antibody test, and/or a diagnosis made by a doctor or nurse. Patients who reported not having a COVID-19 infection or believed they had the infection without undergoing testing were included in the ´No COVID-19 group´ (Fig. 1).

Patients were asked to report the month and year of their first COVID‑19 infection. Based on these data, infections were categorized into time intervals corresponding to the dominant SARS‑CoV‑2 variant at the time, as defined by the Public Health Agency of Sweden^16^: March 2020–February 2021 (Wuhan), March–June 2021 (Alpha), July 2021–December 2021 (Delta), and from January 2022 onward (Omicron). Patients responded whether they were hospitalised due to COVID-19, in a regular ward, or in the intensive care unit (ICU). Self-assessed severity of COVID-19 was categorized into mild (merged ´very mild´ and ´mild´), moderate, and severe (merged ´severe´ and ´very severe´). Vaccination status against COVID-19 was recorded using the following response options: no vaccine, any vaccine (one, two, three, or four doses).

### Post-COVID-19 symptoms

Within the COVID-19 group, patients who reported persistent, new, or worsening symptoms lasting more than three months following their COVID-19 infection were classified as the post-COVID-19 symptoms group. The questionnaire included a multiple-choice question listing the following symptoms: dyspnoea (during light or strenuous exercise), cough/rhinitis, tachycardia, fatigue, myalgia/joint pain, runny nose/nasal congestion, memory/concentration problems, and anxiety/depression. An additional free-text option allowed participants to report other persistent symptoms not listed. The variable for anosmia/dysgeusia was extracted from free-text responses.

### Sociodemographic and clinical characteristics

Data on sex and age were obtained from the questionnaire. Educational level was categorised into low and high levels. A high level of education was defined as having completed more than two years of study beyond the nine years of compulsory schooling. Smoking status was categorized as daily, ex/sometimes, or never. Body mass index was calculated from the weight and height in the questionnaire. Obesity was defined as body mass index (BMI in kg/m^2^) ≥ 30, overweight as BMI ≥ 25 and ≤29.9, and underweight as BMI < 20. Underweight categories were based on the previous studies^17^, and the other categories on the WHO. Comorbidities were assessed using a multiple-choice question: ´Do you have or have you had any of the following diseases? ´. The listed conditions included allergic rhinitis or conjunctivitis, hypertension, anxiety/depression, heart disease, chronic pain, sleep apnoea, and diabetes mellitus.

### Statistical analysis

Basic characteristics of the study patients are presented as means with standard deviation (SD) for continuous variables and as absolute numbers and percentages for categorical variables. The differences between groups are assessed by descriptive methods using t-tests or Pearson’s chi^-^square test to determine p-values. The association between confirmed COVID-19 or post-COVID-19 symptoms and other relevant variables was assessed with logistic regression. The main model was adjusted for age and asthma/COPD (lung diagnosis), and the secondary models were crude and adjusted for age, asthma/COPD, and sex. Age was treated as a categorical variable, grouped into 10-year intervals. The reference group for each variable is presented in supplementary tables 1 and 3. Results from the logistic regression analysis are reported as odds ratios (ORs) with 95% confidence intervals (CIs). To assess the prevalence of different post-COVID-19 symptoms and their interrelationships, we constructed a pairwise co-occurrence matrix. As a sensitivity analysis for the main analysis assessing association between post-COVID-19 and other factors, to account for multiple comparisons, the Benjamini–Hochberg procedure^18^ was applied to p-values from the adjusted logistic regression models to control the false discovery rate at 5%. The correction was performed across the exposure–outcome associations included in this sensitivity analysis.

Missing values were reported and handled using complete-case analysis.

All analyses were performed using STATA/MP 18.0, with p-values of <0.05 considered statistically significant.

### Ethics

The study was approved by the Swedish Ethical Review Authority (Dnr 2004:M-445, Dnr 2011/318, Dnr 2021-03537). All participants provided written informed consent prior to their participation. The study was conducted in accordance with the principles outlined in the revised Declaration of Helsinki.

## Results

### Characteristics

The study population consisted of 2328 patients, of whom 44% had COPD and 56% had asthma (Table 1). Patients with COPD were older than those with asthma. The education level was lower in patients with COPD. Smoking, ex/sometimes, and daily, was more common in patients with COPD. Patients with asthma were more frequently classified as overweight or obese (BMI ≥ 25 kg/m^2^), whereas a greater proportion of those with COPD had a BMI below 20 kg/m^2^.

**Table 1:** Basic characteristics of the study population.

Characteristics	COPD n = 1032 (44%)	Asthma n = 1296 (56%)
**Age, mean ± SD**	75 ± 7	63 ± 14
**Female sex, n (%)**	618 (60)	791 (61)
**BMI groups in kg/m**^**2**^**, n (%)**		
<20	97 (9)	34 (3)
20–24.9	300 (29)	396 (31)
25–29.9	351 (34)	509 (39)
≥30	282 (27)	353 (27)
**Education level, n (%)**		
Lower	767 (76)	624 (49)
Higher	246 (24)	655 (51)
**Smoking, n (%)**		
Current	157 (15)	41 (3)
Ex/sometimes	797 (78)	495 (39)
Never	66 (6)	740 (58)
**Comorbidity, n (%)**		
Allergic rhinitis or conjunctivitis	172 (17)	730 (56)
Hypertension	610 (59)	563 (43)
Anxiety/depression	177 (17)	257 (20)
Heart disease	268 (26)	221 (17)
Chronic pain	158 (15)	225 (17)
Sleep apnoea	141 (14)	128 (10)
Diabetes mellitus	212 (21)	190 (15)

### COVID-19 frequency, characteristics and risk factors

The frequency of confirmed COVID-19 was 22%. Patients in the COVID-19 group were younger compared to the ´No COVID-19 group´ (Table 2). Both groups were similar regarding sex and mean BMI, except that significantly fewer patients with a BMI < 20 had COVID-19. Most patients experienced their first infection from January 2022 onward (55%), followed by March 2020–February 2021 (23%), July 2021–December 2021 (12%), and March–June 2021 (11%).

**Table 2:** Characteristics of study patients with asthma or COPD in the two groups: COVID-19 and No COVID-19.

Characteristics	COVID-19 n = 494	No COVID-19 n = 1758
**Age, mean **± **SD**	59 ± 15	71 ± 11
**Female sex, n (%)**	292 (59)	1069 (61)
**Lung diagnosis, n (%)**		
COPD	120 (24)	872 (50)
Asthma	374 (76)	886 (50)
**BMI groups, kg/m**^**2**^**, n (%)**		
<20	15 (3)	111 (6)
20–24.9	154 (31)	523 (30)
25–29.9	193 (39)	643 (37)
≥30	132 (27)	475 (27)
**Education level, n (%)**		
Lower	220 (45)	1116 (64)
Higher	268 (55)	614 (36)
**Smoking, n (%)**		
Daily	23 (5)	172 (10)
Ex/sometimes	210 (43)	1032 (59)
Never	254 (52)	534 (31)
**Comorbidity, n (%)**		
Allergic rhinitis or conjunctivitis	264 (53)	623 (35)
Hypertension	192 (39)	952 (54)
Anxiety/depression	112 (23)	311 (18)
Heart disease	68 (14)	401 (23)
Chronic pain	79 (16)	291 (17)
Sleep apnoea	50 (10)	212 (12)
Diabetes mellitus	66 (13)	324 (18)
**Self-assessed severity of acute COVID-19, n (%)**		
Mild	295 (61)	-
Moderate	129 (27)	-
Severe	63 (13)	-
**Hospitalised due to acute COVID-19, n (%)**	44 (9)	-

Missing variables: For the COVID-19 column: BMI, N = 12; education, N = 6; Smoking, N = 7; Self-assessed severity of COVID-19, N = 1; For No COVID-19: BMI, N = 59; education, N = 25; Smoking, N = 20. The question on comorbidities and hospitalization due to acute COVID-19 was constructed as a multiple-response question, in which participants were asked to select “Yes” for all comorbidities that applied to them.

Variables associated with COVID-19 infection were identified using logistic regression analysis (Supplementary Table 2). In adjusted analysis, higher age was negatively associated with COVID-19. Asthma was associated with a significantly higher risk of COVID-19 compared to COPD, with an odds ratio of 1.52 (95% CI: 1.18–1.97). In the COVID-19 group, 18 subjects (4%) reported not having any vaccination against COVID-19, and in the ´No COVID-19 group´, there were 32 subjects (2%).

### Post-COVID-19 symptoms and their prevalence

**Fig. 2:**
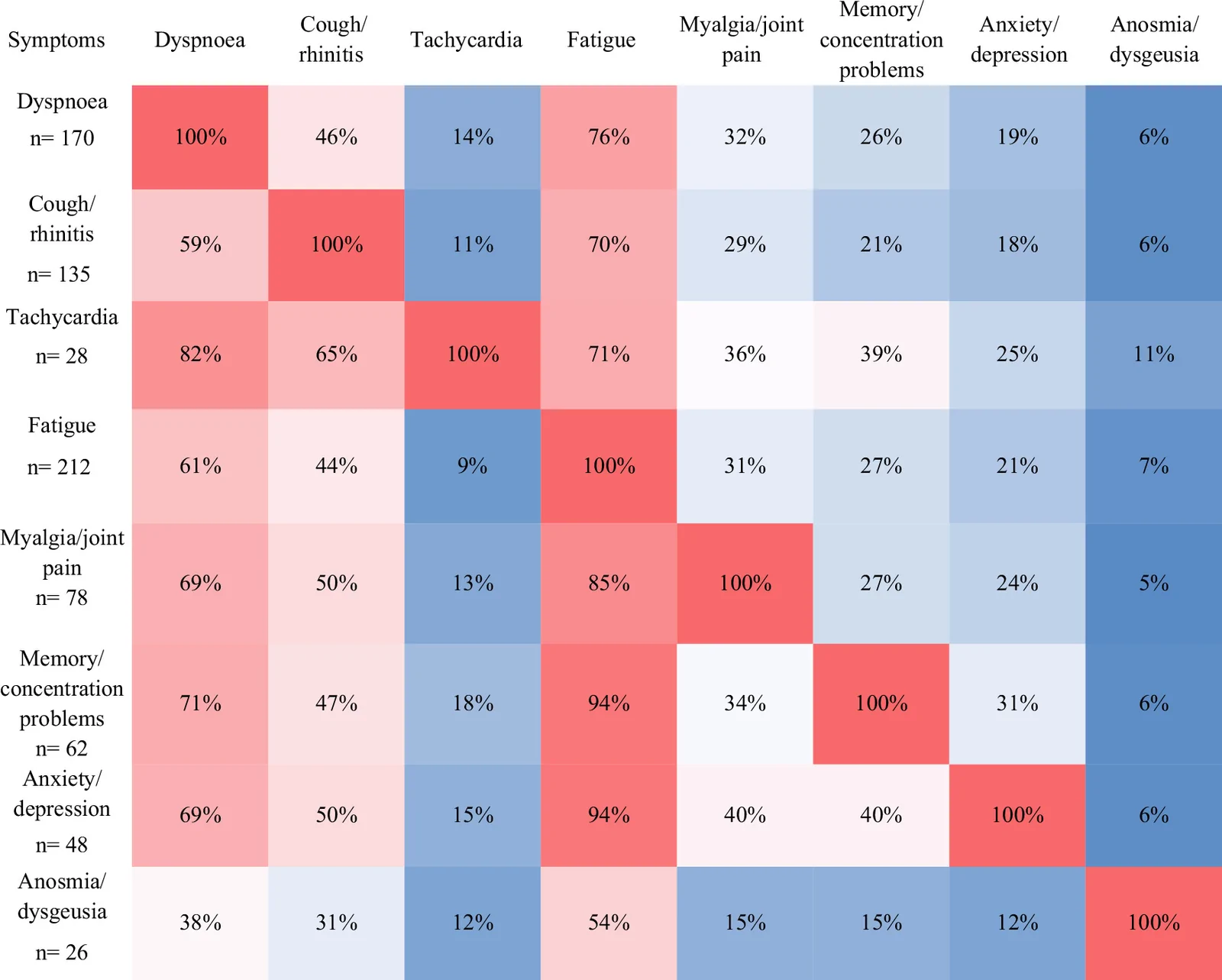
A pairwise co-occurrence matrix of reported symptoms in patients with asthma and COPD (n varies per symptom), visualized as a heatmap. Rows represent symptom X and columns represent symptom Y. Each cell shows the percentage of individuals with symptom X who also report symptom Y. The highest percentages are shown in red, the lowest in blue.

Prevalence of post-COVID-19 symptoms was 289 (59%) within the COVID-19 group. In patients with asthma, 55% reported post-COVID-19 symptoms, and 70% of patients with COPD reported post-COVID-19 symptoms. A total of 147 patients reported 1–2 post-COVID-19 symptoms, while 142 patients reported 3 or more symptoms. The most common post-COVID-19 symptoms were fatigue, dyspnoea, and cough/ rhinitis, in both asthma and COPD (Supplementary Table 3). Patients reporting any symptom, except anosmia or dysgeusia, also reported dyspnoea and fatigue (Fig. 2).

### Post-COVID-19 symptoms characteristics and risk factors

Patients with post-COVID-19 symptoms were older, had a higher frequency of obesity, lower level of education, and were more likely to smoke compared to patients without post-COVID-19 symptoms (Table 3). Although more women had persistent symptoms, the difference was not significant. Patients reporting post-COVID-19 symptoms reported more severe acute COVID-19 infection, with a higher frequency of hospitalisation. Additionally, the post-COVID-19 symptoms group included more patients with a history of COPD, hypertension, anxiety/ depression, stroke, and chronic pain. There were no significant differences in vaccination status between the groups.

**Table 3:** Characteristics of the study patients with and without post-COVID-19 symptoms.

Characteristics	Post-COVID-19 symptoms n = 289 (59%)	No post-COVID-19 symptoms n = 205 (41%)	P-value
**Age, years, mean ± SD**	61 ± 14	56 ± 15	0.0001
**Female, n (%)**	180 (62)	112 (55)	0.088
**Lung diagnosis, n (%)**			
COPD	84 (29)	36 (18)	0.003
Asthma	205 (71)	169 (82)	0.003
**BMI groups, kg/m**^**2**^**, n (%)**			
<20	6 (2)	9 (4)	0.140
20–24.9	75 (26)	79 (39)	0.003
25–29.9	113 (39)	80 (39)	0.986
≥30	95 (33)	37 (18)	<0.0001
**Education level, n (%)**			
Lower	147 (51)	139 (36)	<0.0001
Higher	139 (49)	129 (64)	<0.0001
**Smoking, n (%)**			
Daily	14 (5)	9 (4)	0.011
Ex/sometimes	72 (36)	138 (49)	0.011
Never	122 (60)	132 (47)	0.011
**Comorbidity, n (%)**			
Allergic rhinitis or conjunctivitis	155 (54)	109 (53)	0.919
Hypertension	125 (43)	67 (33)	0.018
Anxiety/depression	75 (26)	37 (18)	0.039
Heart disease	45 (16)	23 (11)	0.167
Chronic pain	65 (23)	14 (7)	<0.0001
Sleep apnoea	33 (11)	17 (8)	0.256
Diabetes mellitus	44 (15)	22 (11)	0.148
**Self-assessed severity of acute COVID-19, n (%)**			
Mild	147 (51)	148 (74)	<0.0001
Moderate	83 (29)	46 (23)	0.161
Severe	58 (20)	5 (3)	<0.0001
**Hospitalised due to acute COVID-19, n (%)**	39 (14)	9 (4)	0.001
**No vaccine against COVID-19, n (%)**	14 (5)	4 (2)	0.096

In the adjusted model for age and lung diagnoses, higher age, female sex, and obesity were associated with an increased likelihood of reporting post-COVID-19 symptoms. The severity of the initial COVID-19 infection had the strongest association with post-COVID-19 symptoms, as reflected by a higher OR for hospitalisation, self-assessed severity, and a lower OR for not seeking medical care (Supplementary Table 4).

**Fig. 3:**
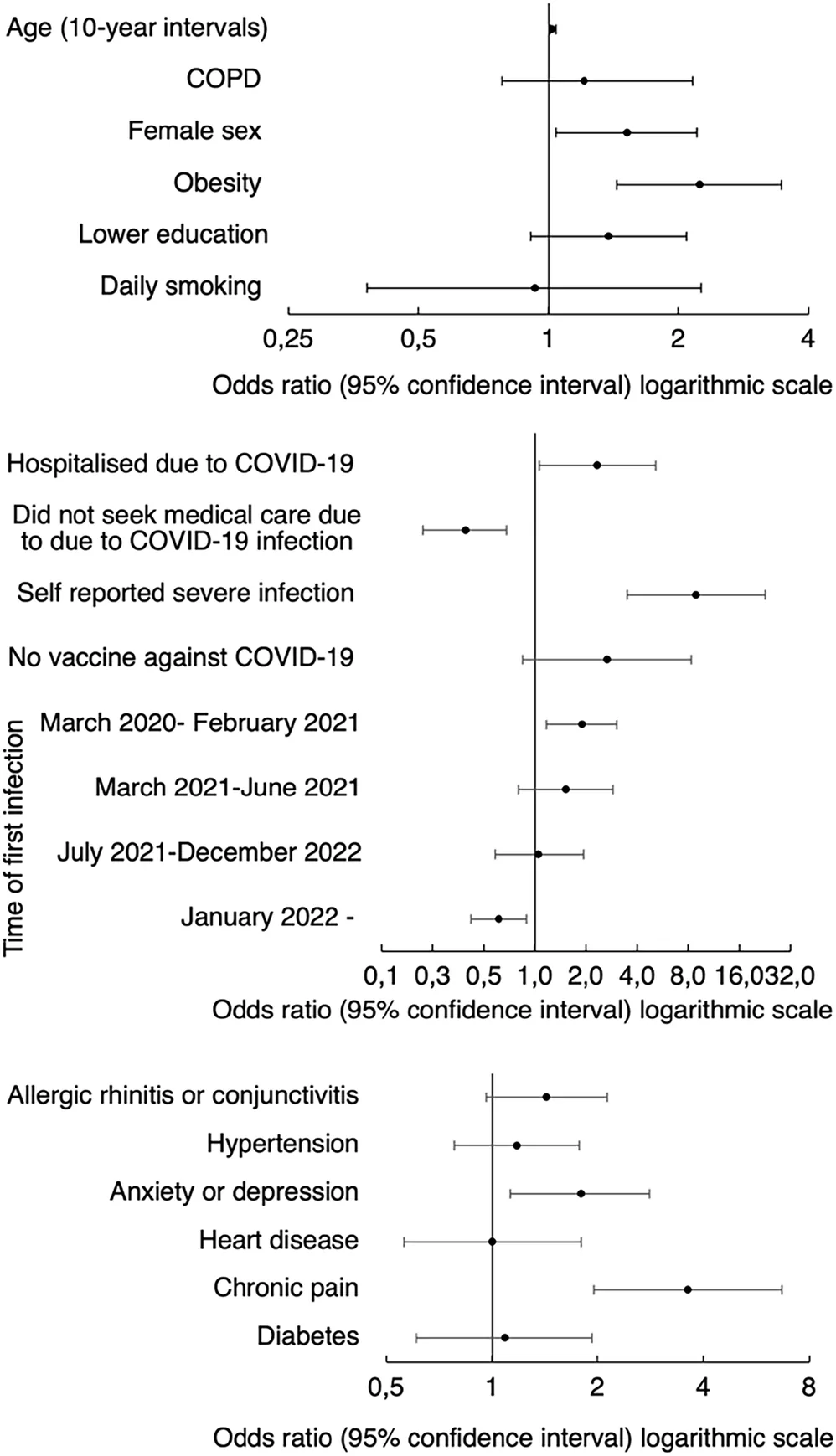
The association between post-COVID-19 symptoms and patients’ characteristics. Associations were assessed by logistic regression with adjustment for age, grouped in 10-year intervals, and lung disease diagnoses (COPD or asthma) presented as odds ratios with 95% confidence intervals. For the comparison between lung disease diagnoses, asthma was the reference category.

In the main adjusted analysis, anxiety/depression and chronic pain significantly increased the odds of post-COVID-19 symptoms. In contrast, hypertension, allergies, heart disease, and diabetes were not associated with post-COVID-19 symptoms after adjusting for age and asthma/COPD. Regarding time of first COVID-19 infection, March 2020–February 2021 was associated with an increased risk of post-COVID-19 symptoms; in contrast, January 2022 onward was significantly associated with a lower risk. COPD was not significantly associated with post-COVID-19 symptoms compared with asthma after adjustment for age (Fig. 3). Supplementary Table 3 also presents ORs additionally adjusted for sex, which did not differ from the ORs adjusted for age and lung diagnosis.

In the sensitivity analysis correcting for multiple comparisons using the Benjamini–Hochberg false discovery rate procedure, severe acute COVID-19 infection, obesity, chronic pain, not seeking medical care during the acute COVID-19 infection, and anxiety/depression remained associated with post-COVID-19 symptoms. Female sex and hospitalization, which were statistically significant in the main adjusted model, did not remain significant after correction for multiple comparisons (Supplementary Table 5).

## Discussion

In this cross-sectional study of 2 328 patients with asthma or COPD from the Swedish PRAXIS cohort, we showed that 22% reported having had confirmed COVID-19 at least once. Asthma was, compared to COPD, associated with a higher risk of COVID-19. Among those infected, 59% experienced post-COVID-19 symptoms. In the main analysis, we found that significant risk factors for post-COVID-19 symptoms were self-reported severity of acute infection, hospitalisation due to the acute infection, higher age, female sex, and obesity, as well as comorbidities including anxiety/depression and chronic pain.

We found that a higher proportion of individuals with asthma reported having confirmed COVID-19 compared to those with COPD. This probably reflects the testing and isolation recommendations during the COVID-19 pandemic in Sweden^19^. In the early stages of the pandemic, testing was limited and primarily focused on healthcare workers and those with more severe symptoms^20^. In contrast, other individuals, particularly older adults with COPD and multiple comorbidities, were recommended to stay at home and avoid exposure to the virus. Consequently, as COPD is more common in older people, fewer of those had confirmed infection.

We showed that 59% of asthma and COPD patients reported persistent, new, or worsening symptoms after COVID-19 infection. Post-COVID-19 symptoms were reported by 55% of asthma patients and 70% of COPD patients. A meta-analysis showed that individuals with pre-existing asthma and COPD had a 41 and 32% increased risk, respectively, of developing post-COVID-19 condition, compared to those without chronic respiratory conditions^13^. In contrast, a retrospective study from the USA based on electronic health records reported that only 2% of patients with obstructive lung disease received a clinical diagnosis of post-COVID-19^21^. This discrepancy may reflect differences in methodology and healthcare-seeking behaviour. Many individuals with persistent symptoms may not seek medical care, especially if symptoms are mild or attributed to their underlying respiratory condition. Additionally, some symptoms may go undocumented in clinical records, leading to underdiagnosis in registry-based studies. Variability in post-COVID-19 prevalence estimates may also stem from differences in definitions, data sources, and timing during different pandemic waves, all of which influence comparability across studies.

Our findings on risk factors for post-COVID-19 symptoms align well with existing knowledge about risk factors for post-COVID-19 in patients with and without obstructive lung diseases. For example, female sex has repeatedly been associated with a higher risk of developing post-COVID-19 symptoms in several studies, a result also observed^11,21–23^, and an association was also observed in our main analysis. However, this association was not statistically significant in the sensitivity analysis, suggesting that the finding should be interpreted with caution. Several mechanisms have been proposed to explain the higher risk observed among women in previous studies. These include differences in androgen activity during the acute phase of infection^24^. Additionally, elevated SARS-CoV-2 IgG antibody levels in women during the acute phase have been proposed as a contributing factor to the risk of post-COVID-19 condition^25^. Another biological mechanism linked to post-COVID-19 in women involves uncontrolled type-I interferon signalling and type I interferonopathy^26^. However, a Swiss study on post-COVID-19 condition highlighted that sociocultural factors, rather than biological sex alone, serve as key risk factors of post-COVID-19 and may partially account for the increased risk observed in women^27^.

Higher age has been identified as a risk factor for post-COVID-19 symptoms in some studies^21,22,28^, including our current study, while others have reported the opposite relationship^29^. This discrepancy may be explained by the fact that many older patients did not survive their COVID-19 infection, and the ones who did are more likely to experience lingering consequences^22^. Conversely, studies suggesting older age as a protective factor often attribute this to the higher vaccination rates among the older population, as complete vaccination has been found to reduce the risk of post-COVID-19^30^.

We observed that obesity was a risk factor for post-COVID-19 symptoms, and this aligns with numerous studies^21,31–33^. A Swedish study found no cardiovascular or metabolic chronic conditions as a risk factor for post-COVID-19 diagnosis^5^. One possible explanation for the increased risk is the presence of chronic inflammation and metabolic dysfunction, often observed in patients with obesity^34^.

Our study suggests that self-reported anxiety or/and depression are linked to a higher risk of developing post-COVID-19 symptoms, consistent with findings from other studies^21,22,35^. Possible mechanisms include chronic systemic inflammation and dysregulation of the hypothalamic–pituitary–adrenal axis^36^. Also, having chronic pain is a risk factor for post-COVID-19 symptoms in various other studies^29,37^. Chronic pain was also a risk factor for reporting persistent symptoms after infection with influenza^38^.

Our results indicate that patients reported having their first infection March 2020–February 2021, when the Wuhan variant was dominant, compared with those first infected March 2021 onward, had a higher risk of developing post-COVID-19 symptoms. This finding is consistent with previous studies^39,40^. This could be explained as an effect of vaccination, increasing immunity in the population, and better clinical management of acute infection in the later phases of the pandemic^30^.

### Strengths and limitations

A strength of our study lies in including a randomly selected population from both primary and secondary care settings, enhancing its generalizability. The study covers a broad range of COVID-19 infection severities. Importantly, not all participants sought medical care for their acute infection or post-COVID-19 symptoms, which reduces selection bias, often associated with registry-based studies, and provides a more comprehensive view of the population.

However, there are notable limitations. We did not have information on whether the symptoms had an impact on everyday functioning, which is one of the criteria in the WHO’s definition of post-COVID-19 condition^41^. As the data is self-reported, there is a potential for recall bias, particularly regarding the timing of the first infection, as participants were asked to recall events up to two years after the start of the pandemic. We could not draw a definitive conclusion about the relationship between strain variants and post-COVID-19 symptoms, as the questions regarding post-COVID-19 symptoms were not explicitly linked to the timing of the first reported infection. Finally, the number of patients with COVID-19 and post-COVID-19 symptoms was relatively small, which did not allow adjustment beyond the basic confounders included in the main model. Another limitation is the inability to establish causal relationships due to the cross-sectional design of the study.

### Clinical relevance

The results of this study may contribute to identifying patients who should be prioritized for preventive measures in future viral pandemics. Preventive measures, including vaccines for individuals at higher risk of developing post-COVID-19 symptoms, may decrease the likelihood of these outcomes^42^. Additionally, the identified risk factors can guide care for patients with chronic airway diseases and help prioritize them for rehabilitation following viral infections.

### Conclusion

In our study, the prevalence of post-COVID-19 symptoms in patients with asthma and COPD was 59%, which was high compared to other studies. Our analysis suggests several demographic, clinical, and comorbid) risk factors for post-COVID-19 symptoms, which can help identify patients at higher risk.

## Supplementary information

Supplementary Information

## Data Availability

The datasets used and analyzed in this study are accessible for collaboration with the research team.
