# Supplementary material for: Risk factors of post-COVID-19 symptoms, a cross-sectional study in patients with asthma and chronic obstructive lung disease
Source: NPJ Prim Care Respir Med. 2026 Sep 29;36:64. doi: 10.1038/s41533-026-00570-x (PMC13620127; doi:10.1038/s41533-026-00570-x)
Supplement: Supplementary file 1 [file 41533_2026_570_MOESM1_ESM.docx]

**Supplementary Figure 1:** Flow chart describing the origin of the study population.

# Supplementary Table 1: Patient questionnaire - Asthma and COPD. This excerpt is from the PRAXIS 2022 questionnaire, which provided the data for the study. More information about PRAXIS and full questionnaires are available on https://praxisstudien.com/en/

| **Question**  **Asthma** | **Question**  **COPD** | **Question / Response options** |
| --- | --- | --- |
| 1 | 1 | **How old are you?** ____ years |
| 2 | 2 | **Are you?** ☐ Female ☐ Male |
| 3 | 6 | **Do you have, or have you had, any of the following conditions?** *(Tick all that apply.)* ☐ High blood pressure☐ Heart disease ☐ Stroke ☐ Chronic pain ☐ Polymyalgia rheumatica (PMR) ☐ Claudication (intermittent claudication) ☐ Crohn’s disease or ulcerative colitis ☐ Lung cancer (only in COPD) ☐ Anxiety/Depression ☐ Rheumatic joint disease ☐ Osteoporosis ☐ Allergic rhinitis or conjunctivitis ☐ Sleep apnoea ☐ Diabetes ☐ None of these |
| 16 | 17 | **Have you had Covid-19 (Coronavirus)?** *(Multiple answers possible.)* ☐ Yes, confirmed by a throat/nasal swab (PCR test) ☐ Yes, confirmed by a rapid test (nasal swab) ☐ Yes, confirmed by an antibody test (blood test) ☐ Yes, according to a doctor or nurse☐ Yes, I believe so because my symptoms were typical of Covid-19, but I was not tested ☐ No, I do not think so.  *If No, continue to Question 21 (22 in COPD). If Yes, answer Questions 17–20 below (18-20 in asthma)* |
| 17 | 18 | **When did you develop Covid-19?** *(If more than once, indicate the first occasion. State month and year.)* Became ill in _____ month, Year _____ |
| 18 |  | **Have you been admitted to hospital or sought emergency medical care anywhere because of Covid-19 (Coronavirus)?** *(Multiple answers possible.)* ☐ Yes, sought urgent care at a GP surgery/out-of-hours clinic ☐ Yes, sought emergency care at a hospital ☐ Yes, admitted to a hospital ward☐ Yes, admitted to an intensive care unit☐ No, neither sought emergency care nor been hospitalised |
|  | 19 | **Have you been admitted to hospital or sought emergency medical care anywhere because of Covid-19 (Coronavirus)?** *(Multiple answers possible.)* ☐ Yes, sought emergency care ☐ Yes, admitted to a hospital ward☐ Yes, admitted to an intensive care unit☐ No, neither sought emergency care nor been hospitalised |
| 19 | 20 | **How would you describe the severity of your Covid-19 infection?** ☐ Very mild ☐ Mild ☐ Moderately severe ☐ Severe ☐ Very severe |
| 20 | 21 | **If you still have, or had, persistent symptoms (longer than 3 months) following your Covid-19 infection, which of the following symptoms do you feel appeared or increased compared with before you had Covid-19?** *(Multiple answers possible.)*☐ Shortness of breath during strenuous exercise (dyspnoea)☐ Shortness of breath during light exercise (dyspnoea)☐ Cough☐ Tachycardia☐ Fatigue☐ Myalgia/joint pain☐ Rhinitis☐ Memory or concentration problems☐ Anxiety/depression☐ Other: __________ |
| 63 | 72 | **Have you been vaccinated against Covid-19 (Coronavirus)?**☐ Yes, one dose☐ Yes, two doses☐ Yes, three doses☐ Yes, four doses☐ No |
| 65 | 75 | **How tall are you?**_____ cm |
| 66 | 76 | **How much do you weigh?**_____ kg |
| 67 | 77 | **What is the highest level of education you have completed?** ☐ Less than 5 years of schooling☐ Primary or compulsory school☐ Lower secondary school or girls’ school☐ Two-year upper secondary school or vocational school☐ Three- to four-year upper secondary school☐ University/college, 2.5 years or less☐ University/college, 3 years or more |
| 72 | 82 | **Do you smoke?** ☐ No, I have never smoked regularly☐ No, I have stopped smoking☐ Yes, I smoke sometimes☐ Yes, I smoke daily |

**Supplementary Table 2**: Logistic regression for COVID-19 infection

| Characteristic (reference group) | N participants for both models | Not adjusted  OR (95% CI) | Adjuted for age and asthma /COPD  OR (95% CI) |
| --- | --- | --- | --- |
| Age (10-year intervals) | 2,252 | 0.53 (0.49-0.57) | 0.56 (0.52-0.61) |
| Asthma (COPD) | 2,252 | 3.06 (2.45-3.84) | 1.52 (1.18-1.97) |
| Female (male) | 2,252 | 0.93 (0.76-1.14) | 0.80 (0.64-1.00) |
| Obesity (no obesity) | 2,252 | 0.98 (0.79-1.23) | 0.92 (0.72-1.17) |
| Lower education (higher) | 2,221 | 0.45 (0.37-0.56) | 0.94 (0.74-1.20) |
| Daily smoking (no daily smoking) | 2,252 | 0.45 (0.29-0.71) | 0.61 (0.38-0.98) |
| Allergy (no allergy) | 2,252 | 2.09 (1.71-2.56) | 1.11 (0.88-1.41) |
| Hypertension (no hypertension) | 2,252 | 0.53 (0.44-0.66) | 0.94 (0.75-1.19) |
| Anxiety /depression (no anxiety /depression) | 2,252 | 1.36 (1.07-1.74) | 0.98 (0.75-1.28) |
| Heart disease (no heart disease ) | 2,252 | 0.54 (0.41-0.71) | 1.00 (0.74-1.35) |
| Diabetes (no diabetes) | 2,252 | 0.68 (0.51-0.91) | 0.99 (0.73-1.34) |
| No vaccine against COVID-19 (vaccine against COVID-19) | 2,228 | 1.81 (1.02-3.22) | 1.46 (0.78-2.74) |

Notes: Since there was no missing data in age, and asthma/COPD variables, the number of participants applies to all three models.

**Supplementary Table 3**: Prevalence of different symptoms within the COVID-19 group

| Symptoms | Dyspnea | Cough/ rhinitis | Tachycardia | Fatigue | Myalgia/joint pain | Memory/ concentration problems | Anxiety/ depression | Anosmia/ dysgeusia |
| --- | --- | --- | --- | --- | --- | --- | --- | --- |
| COPD  n (%) | 50 (42) | 48 (40) | 9 (8) | 63 (53) | 26 (22) | 18 (15) | 15 (13) | 6 (5) |
| Asthma  n (%) | 120 (32) | 87 (23) | 19 (5) | 149 (40) | 52 (14) | 44 (12) | 33 (9) | 20 (5) |

**Supplementary Table 4:** Logistic regression for post-COVID-19 symptoms

| Characteristics (reference group) | N participants for all models | Not adjusted  OR (95% CI) | Adjusted for age and asthma /COPD  OR (95% CI) | Adjusted for age, asthma /COPD and female/male sex  OR (95% CI) |
| --- | --- | --- | --- | --- |
| Age (10-year intervals) | **494** | 1.30 (1.15-1.48) | 1.25 (1.09-1.45) | 1.28 (1.11-1.49) |
| COPD (asthma) | **—** | 1.92 (1.24-2.99) | 1.30 (0.78-2.16) | 1.27 (0.76-2.11) |
| Female (male) | **494** | 1.37 (0.95-1.97) | 1.52 (1.04-2.21) | 1.52 (1.04-2.21) |
| Obesity (no obesity) | **494** | 2.22 (1.44-3.43) | 2.24 (1.44-3.47) | 2.16 (1.39-3.37) |
| Lower education (higher) | **488** | 1.86 (1.29-2.70) | 1.38 (0.91-2.09) | 1.41 (0.93-2.15) |
| Daily smoking (no daily smoking) | **487** | 1.12 (0.47-2.63) | 0.93 (0.38-2.26) | 0.95 (0.39-2.31) |
| Hospitalized due to COVID-19 infection  (not hospitalized due to COVID-19 infection) | **494** | 3.40 (1.61-7.18) | 2.33 (1.06-5.12) | 2.27 (1.03-5.01) |
| Did not seek medical care due to due to COVID-19  (Did seek medical care due to due to COVID-19) | **494** | 0.31 (0.18-0.54) | 0.39 (0.22-0.68) | 0.40 (0.23-0.70) |
| Severe acute COVID-19 (mild or moderate COVID-19) | **487** | 9.78 (3.85-24.88) | 8.91 (3.49-22.77) | 9.00 (3.50-23.00) |
| Allergy (no allergy) | **494** | 1.02 (0.71-1.46) | 1.43 (0.96-2.13) | 1.44 (0.96-2.15) |
| Hypertension (no hypertension) | **494** | 1.57 (1.08-2.28) | 1.18 (0.78-1.77) | 1.19 (0.80-1.80) |
| Anxiety /depression (no anxiey/ depression) | **494** | 1.59 (1.02-2.48) | 1.79 (1.13-2.80) | 1.68 (1.06-2.66) |
| Heart disease (no heart disease) | **494** | 1.46 (0.85-2.50) | 1.00 (0.56-1.79) | 1.00 (0.56-1.79) |
| Chronic pain (no chronic pain) | **494** | 3.96 (2.15-7.28) | 3.61 (1.95-6.69) | 3.47 (1.87-6.43) |
| Diabetes (no diabetes) | **494** | 1.49 (0.86.2.58) | 1.09 (0.61-1.93) | 1.15 (0.65-2.06) |
| No vaccine against COVID-19  (vaccine against COVID-19) | **488** | 2.53 (0.82-7-79) | 2.67 (0.85-8.34) | 2.42 (0.77-7.62) |
| Alpha (Delta and Omikron)* | 368 | 1.78 (1.22-2.59) | 1.64 (1.12-2.40) | 1.68 (1.15-2.48) |
| Delta (Alpha and Omikorn)* | 368 | 1.15 (0.64-2.08) | 1.05 (0.57-1.92) | 1.08 (0.59-1.99) |
| Omikron (Alpha and Delta)* | 368 | 0.55 (0.38-0.79) | 0.61 (0.42-0.89) | 1.59 (0.40-0.86) |

Notes: Since there was no missing data in age, sex, and asthma/COPD variables, the number of participants applies to all three models. *Missing date of infection N = **126.**

**Supplementary Table 5.** Sensitivity analysis using the Benjamini–Hochberg false discovery rate correction for multiple comparisons

| **Characteristic (reference)** | **p-value *** | **p-value FDR adjusted** |
| --- | --- | --- |
| Severe acute COVID-19 | **0.0005** | **0.0023** |
| Obesity | **0.0005** | **0.0023** |
| Chronic pain | **0.0005** | **0.0023** |
| Did not seek medical care | **0.0010** | **0.0035** |
| Anxiety/depression | **0.0130** | **0.0364** |
| Female sex | **0.0300** | 0.0660 |
| Hospitalised | **0.0330** | 0.0660 |
| Allergic rhinitis or conjunctivitis | 0.0970 | 0.1498 |
| No COVID-19 vaccine | 0.0990 | 0.1498 |
| Education | 0.1070 | 0.1498 |
| Hypertension | 0.3910 | 0.4976 |
| Diabetes | 0.6920 | 0.8073 |
| Daily smoking | 0.8720 | 0.9391 |
| Heart disease | 0.9520 | 0.9520 |

Notes: Benjamini–Hochberg false discovery rate (FDR) correction was applied to p-values from the adjusted logistic regression analyses (see Figure 3*). An FDR-adjusted p-value <0.05 was considered statistically significant. significant_fdr = 1 indicates statistical significance after FDR correction, whereas significant_fdr = 0 indicates that the association did not remain statistically significant after correction.
